# *Owenia fusiformis* – a basally branching annelid suitable for studying ancestral features of annelid neural development

**DOI:** 10.1186/s12862-016-0690-4

**Published:** 2016-06-16

**Authors:** Conrad Helm, Oliver Vöcking, Ioannis Kourtesis, Harald Hausen

**Affiliations:** Sars - International Centre for Marine Molecular Biology, University of Bergen, Thormøhlensgt. 55, Bergen, N-5008 Norway

**Keywords:** Nervous system, Neuroanatomy, Annelida, clsm, Immunohistochemistry, Evolution

## Abstract

**Background:**

Comparative investigations on bilaterian neurogenesis shed light on conserved developmental mechanisms across taxa. With respect to annelids, most studies focus on taxa deeply nested within the annelid tree, while investigations on early branching groups are almost lacking. According to recent phylogenomic data on annelid evolution Oweniidae represent one of the basally branching annelid clades. Oweniids are thought to exhibit several plesiomorphic characters, but are scarcely studied - a fact that might be caused by the unique morphology and unusual metamorphosis of the mitraria larva, which seems to be hardly comparable to other annelid larva. In our study, we compare the development of oweniid neuroarchitecture with that of other annelids aimed to figure out whether oweniids may represent suitable study subjects to unravel ancestral patterns of annelid neural development. Our study provides the first data on nervous system development in basally branching annelids.

**Results:**

Based on histology, electron microscopy and immunohistochemical investigations we show that development and metamorphosis of the mitraria larva has many parallels to other annelids irrespective of the drastic changes in body shape during metamorphosis. Such significant changes ensuing metamorphosis are mainly from diminution of a huge larval blastocoel and not from major restructuring of body organization. The larval nervous system features a prominent apical organ formed by flask-shaped perikarya and circumesophageal connectives that interconnect the apical and trunk nervous systems, in addition to serially arranged clusters of perikarya showing 5-HT-LIR in the ventral nerve cord, and lateral nerves. Both 5-HT-LIR and FMRFamide-LIR are present in a distinct nerve ring underlying the equatorial ciliary band. The connections arising from these cells innervate the circumesophageal connectives as well as the larval brain via dorsal and ventral neurites. Notably, no distinct somata with 5-HT -LIR in the apical organ are detectable in the larval stages of *Owenia*.

Most of the larval neural elements including parts of the apical organ are preserved during metamorphosis and contribute to the juvenile nervous system.

**Conclusions:**

Our studies in *Owenia fusiformis* strongly support that early branching annelids are comparable to other annelids with regard to larval neuroanatomy and formation of the juvenile nervous system. Therefore, *Owenia fusiformis* turns out to be a valuable study subject for comparative investigations and unravelling ancestral processes in neural development in Annelida and Bilateria in general.

**Electronic supplementary material:**

The online version of this article (doi:10.1186/s12862-016-0690-4) contains supplementary material, which is available to authorized users.

## Background

The structure and formation of metazoan nervous systems are topics with long scientific histories, and that relate to basic questions of animal evolution and development. Numerous recent studies have broadened the knowledge on neuroanatomical plasticity and neural patterning mechanisms in several invertebrate groups [1–5]. A major focus of these studies has been to resolve the origin of bilaterian brain structures and trunk nerve cords, and to clarify whether they evolved out of orthogon-like versus net-like nervous systems [4, 6, 7]. In many of these studies annelids have been an important group as they have provided deep insights into the molecular patterning of nervous system development [2, 8, 9], neural circuitry [10] as well as molecular characteristics and functions of specific sensory cells or brain areas in protostomes other than arthropods or nematodes [11, 12]. This has had a significant impact on the general understanding of animal nervous system evolution and function. However, phylogenomic analyses of annelids indicate that thoroughly investigated species such as *Platynereis dumerilii* and *Capitella teleta* are deeply nested at different positions within the annelid tree [13–16], which coincide with different sets of traits relating to the molecular control of neural development. Further, neuroanatomy and the course of neurogenesis have been shown to be variable in annelids. Based on comparative histological and immunohistochemical studies special attention was given to brain complexity and organization, trunk nervous system architecture and centralization, sensory systems and direction of differentiation and maintenance of larval neuronal elements, in the adults [9, 17–31]. Comparative data on nervous system development of the basal-most branching annelid taxa are scarce and are purely based on old histological investigations [32, 33]. Thus, generation of conclusive data is important to provide a basis for studying the organization and developmental patterning of the ancestral annelid nervous system as well as the emergence of nervous system variety and complexity within the group. Oweniids, which occupy the basal-most branch of the annelid tree in recent analyses [14–16] are known to exhibit characters often considered to represent an ancestral condition. Some of these characters are monociliated epidermal cells [34, 35], nephridia similar to those of deuterostomes [36], and a rather simple organized intraepithelial nervous system [37, 38]. Furthermore, certain oweniid species occur in high abundance in the intertidal, and high quantities of larvae can easily be cultured in the lab - an attractive feature for subjects of molecular and developmental studies. However, oweniids have an enigmatic type of larva - the mitraria, which, in contrast to other annelids, undergoes a rather catastrophic metamorphosis [32, 39]. In this study we generated immunohistochemical and histological data to analyze the neuroanatomy of the oweniid *Owenia fusiformis* Delle Chiaje, 1844 from early larva through metamorphosis until the juvenile stage. Our main focus was on whether the larval nervous system is comparable to that of other annelids and whether the main parts of the central nervous system are maintained throughout metamorphosis.

Using this approach we aim to elucidate the oweniid neural development, discuss the ontogeny of adult neuronal precursors, and shed light on the metamorphosis of the remarkable mitraria. Our study points out the potential of *Owenia fusiformis,* one of the basal-most annelid groups, to serve as a valuable model for studying the development, ancestral features, and evolution of the annelid nervous system.

## Results and discussion

### General development of the mitraria

**Fig. 1:**
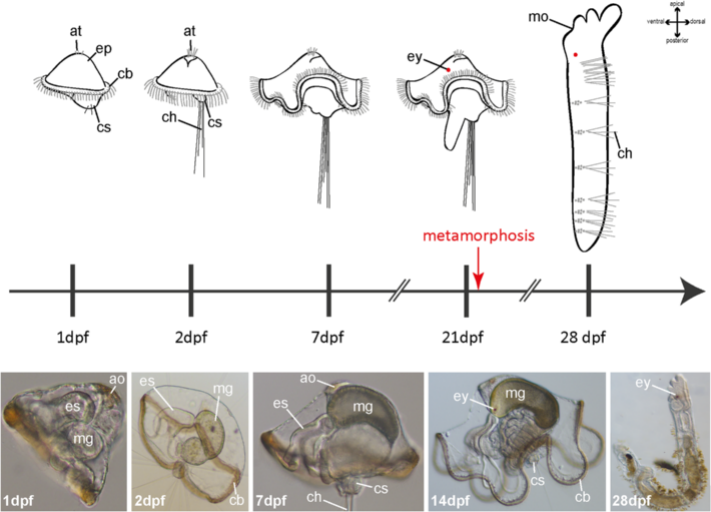
Schematic overview of the normal development of *Owenia fusiformis.* Different developmental stages used in this study are shown in schematic drawings (upper part) and photographs (lower part). The timescale indicates the age of different larval stages according to the emergence of distinct morphological features. Stages are shown in dpf (days post fertilization). Note that the development is somewhat asynchronous and only approximately 80–90 % of the larvae of a certain age exhibit the same developmental stage. ao, apical organ; at, apical tuft; cb, ciliary band; ch, chaetae; cs, chaetal sac; ep, episphere; es, esophagus; ey, eye; mg, midgut; mo, mouth opening

The external features of different developmental stages and early cleavage in Oweniidae have already been examined in detail for *O. fusiformis* and *O. collaris* elsewhere [32, 39]. A synopsis of the development of *O. fusiformis* based on Wilson 1932 [32] and on our own observations is given in the text below, and is shown in Fig. 1. Notably, the developmental speed of the larvae is highly variable within and across batches (largely depending on larval density and food supply). Hence, the timing of development is an approximation. The data shown in this study is based on animals fertilized and reared under lab conditions. Available data to compare the timing of the developmental steps in ‘wild’ animals and specimens reared in the lab are missing so far. Nevertheless, all observed stages refer to stages that can be found under natural conditions, as well.

Reared at 18 °C, the larvae start swimming approximately 24 h after fertilization. At this stage a circumferential band of cilia, the chaetal sacs containing two pairs of short chaetae, an early apical tuft, a mouth and an anus are well developed. Within 48 h post fertilization, the larval episphere is enlarged, the chaetae are elongated, and the larvae start to feed. In the next 3–7 days the characteristic bell-shaped mitraria is almost fully developed featuring the well-developed chaetal sacs with numerous chaetae, a dense ciliated band running around the whole equatorial midline, a through gut, with discernable mouth, esophagus, midgut, hindgut and anus, and a well-developed apical tuft. Solely the eyespots are still missing at this time and appear approximately at 14 dpf (days post fertilization). Posterior of the circumferential ciliary band, the larval hyposphere elongates and forms a worm-shaped appendage. Furthermore, the esophagus and intestine become well developed, and the mid- and hindgut tissue drastically increase in length. At 21 dpf larvae start to metamorphose. This stage is characterized by collapse and oral assimilation of the bell-shaped anterior part of the larva and simultaneous posterior extension of the trunk. Subsequently the chaetae fall off and the chaetal sacs invaginate. Although the body shape changes drastically, the whole metamorphosis can end in just a few minutes.

Annelids in general show a variety of larval types and developmental modes [40], however, oweniid development seems to stand out due to the conspicuous bell-like shape of the larva as well as due to the abrupt and drastic changes in body shape during metamorphosis. In order to better understand the changes in body architecture during metamorphosis of *O. fusiformis*, we firstly investigated the internal anatomy of the free swimming mitraria by serial semi-thin sections, followed by 3D reconstruction and transmission electron microscopy (TEM).

**Fig. 2:**
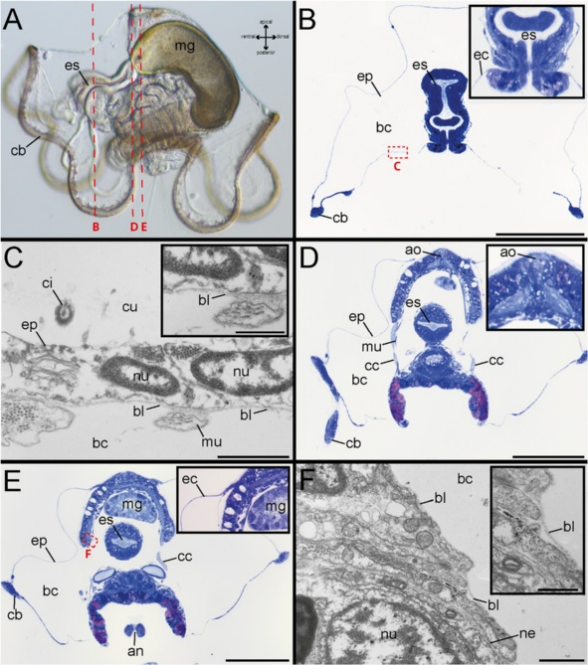
Whole mounts, histological cross-sections and electron microscopical images of *O. fusiformis* at 7 dpf. Light microscopic images of entire larvae (**a**), toluidin-blue stained cross-sections (**b, d, e**) and electron microscopy (**c**, **f**). Note that the sectional planes of b, d, e are indicated by red lines in a. There are three planes. The position of the electron microscopical detail is indicated by red rectangles. **a** Early developmental stage 7 dpf. Ventral (including the mouth opening) is left, apical is up. The animal exhibits a well-developed ciliary band (cb), a distinct esophagus (es) and a defined midgut (mg). **b** In cross-sections of the esophagus region the prominent blastocoel is visible, which occupies most of the space within the larval episphere. The inset shows the esophageal region (es) and the position of the epidermis (ep) in higher magnification. **c** The outer epidermis (ep) is composed of a monolayer of epidermal cells and a prominent cuticle (cu). The underlying basal lamina (bl) borders directly borders the large blastocoel (bc) being a primary body cavity. The inset in (**c**) shows a higher magnification of the basal lamina (bl). **d** A cross-section in the plane of the apical organ (ao) reveals the presence of distinct muscle bundles (mu) and the circumesophageal connective (cc) framing the esophagus (es). The inset shows the the apical organ (ao) in higher magnification. **e** The blastocoel (bc) occupies most of the larva. The inset in (**e**) shows the epidermis (ep) lateral to the apical organ. **f** The central tissue is covered by a basal lamina directly bordering the internal blastocoel (bc). The inset in (**f**) shows a higher magnification of the basal lamina (bl). an, anus; ao, apical organ; bc, blastocoel; bl, basal lamina; cb, ciliary band; cc, circumesophageal connective; ci, cilium; cu, cuticle; ep, epidermis; es, esophagus;; hg, hindgut; mg, midgut; mu, muscle bundle; ne, nerve; nu, nucleus; tr, trunk region. Scale bars = 100 μm (**b**, **d**, **e**), 1 μm (**c**, **f**) and 500 nm (inset in **c** and **f**). The scale bars only refer to the main images in **b**, **d** and **e**, not to the insets

The investigated stage (7 dpf) exhibit a well-developed ciliary band, a distinct esophagus and a midgut (Fig. 2a), which represent the position of the developing juvenile trunk. The region between epidermis and the central trunk tissue is occupied by a very prominent body cavity (Fig. 2b, d and e). Using TEM it became obvious that the epidermis is composed of a thin monolayer of epithelial cells covered by an external cuticle and underlain by a prominent basal lamina, which directly borders the enormous body cavity (Fig. 2c). Similarly, all central tissue, as well as muscle fibres and nerves traversing the body cavity are covered by a basal lamina directly bordering the body cavity (Fig. 2f). This situation defines the body cavity as being a primary body cavity and not a true coelom, which by definition is lined by an epithelium. In conformity with Wilson [32] and Smart and von Dassow [39], we consider the body cavity as a persisting blastocoel and we use this term throughout this investigation. Hence, in larva of *O. fusiformis* the epidermis is noteworthy detached in large areas from underlying mesodermal tissue by the blastocoel. At the level of the apical tuft, a prominent tissue complex underlies the tuft and harbors distinct muscle bundles and circumesophageal connectives, which frame the esophagus, traverse the blastocoel, and contact the posterior area of the curved trunk tissue (Fig. 2b, d and e). Starting from the apical tuft, the epidermis runs down the apical tissue complex until the aforementioned muscle strands emerge into the bastocoel. Here, the epidermis folds backwards and forms the lateral surface of the larva. Hence, the unusual shape of the oweniid swimming larva mainly results from a long persisting and enlarged blastocoel separating the lateral epidermis from the main portions of the developing trunk tissue. Instead, the juvenile morphology is characterized by a well-defined anterior head region and an elongated trunk with numerous chaetae-bearing segments. Interestingly, no blastocoel-like cavities are recognizable in the juvenile worm anymore, although the entire transformation from the mitraria into the post-metamorphic worm takes place within a few minutes. As supported by the detailed observations described in Wilson (1932) [32], we noticed a) anterior uptake of epidermal tissue and b) hypospheric eversion of the juvenile trunk during metamorphosis. Based on our histological data, the drastic change in shape has to result from diminution of the blastocoel and a rearrangement of the lateral detached epidermis and not from a considerable reorganization of the whole body architecture. To what extend histolysis, as described in Wilson (1932), plays a role in these rearrangement processes, needs further investigation and cannot be clarified by the presented data. Nevertheless, the oral invagination of epidermis and the evagination of the trunk at the posterior pole are supported by our own as well as by Wilson’s observations (1932) [32] and by our immunohistochemical data (see further below). As has also been observed by Wilson [32], the tissue harboring the larval apical tuft gets firmly connected to the trunk tissue following metamorphosis and both parts are covered by a continuous epidermis. By comparing the anatomy of pre- and post-metamorphic stages it is likely that the lateral larval epidermis contributes to this continuous epithelium, but this needs further experimental approval. The attachment of the larval apical tissue to the trunk tissue during metamorphosis points towards a continuity of all main body parts during development, from the very apical to the trunk, as it is usual in other annelids. Our immunohistochemical data (see below) show that this continuity is also reflected by the persistence of apical larval neural elements in post-metamorphic stages.

Relative to *Owenia*, clear differences are apparent with respect to the development of certain nemertines, which feature a so-called pilidium larva - the only kind of lophotrochozoan larva that superficially resembles the oweniid mitraria - due to a likewise bell-shaped envelope. Notably, nemertines represent a taxon with the potential of being the annelid sister group [14–16]. In contrast to other nemertines and also to oweniids, body organization of nemertines with a pilidium changes drastically during metamorphosis. In pilidium larvae, the whole anlage of the juvenile forms as a distinct part of the larva. During metamorphosis the juvenile anlage erupts from and subsequently devours the entire remaining larval tissue. Nothing similar is observable in *Owenia*. The main part of the larval tissue gives rise to the juvenile and only part of the epidermis invaginates into the mouth. Although both the oweniid mitraria and the nemertean represent large bell-shaped swimming stages, which differ in shape considerably from the juveniles, the pillidium and the oweniid mitraria have to be regarded as convergently evolved spiralian larval types [41–43].

### Development of early brain precursors and the anterior stomatogastric nervous system

#### Development of the early mitraria (1 dpf- 7 dpf)

**Fig. 3:**
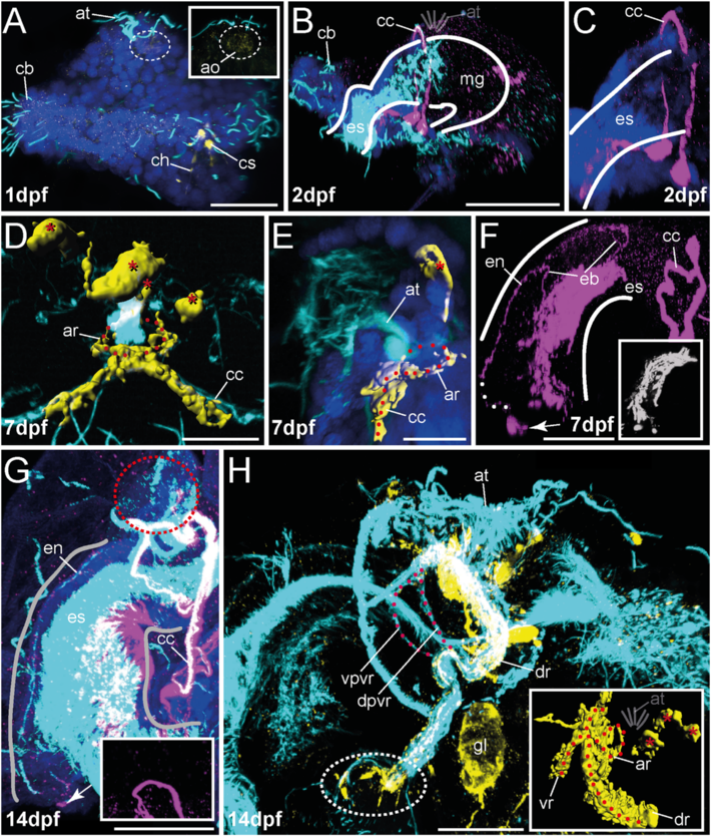
Innervation of apical organ and esophageal region in early developmental stages of *O. fusiformis* (1 dpf - 14 dpf). FMRFamide-LIR (*yellow*) and 5-HT-LIR (*purple*), aTub-LIR (*cyan*), DNA (*dark blue*)**.** Confocal z-projections and volume renderings of FMRFamide-LIR (D, E, H (inset)). Ventral is left and apical is up in all images, except (**d**) which shows a ventral view. Stages are given in days post fertilization (dpf). The shape of the esophagus is indicated by white lines. **a** Early stages exhibit weak FMRFamide-LIR in the apical organ (ao) (white dotted circle). The inset shows the apical organ (ao) (white dotted circle) in higher magnification. Note that the staining in the chaetal sacs (cs) and the chaetae (ch) is due to autofluorescence. The yellow signal along the ciliary band (cb) is caused by unspecific staining and differed between the investigated specimens. The inset shows the FMRFamide-LIR underlying the apical organ (white dotted circle) in higher magnification. **b, c** At 2 dpf the mitraria shows strong 5-HT-LIR in the circumesophageal connective (cc) and in the tissue underlying the esophagus (es). No 5-HT-LIR is visible underlying the apical tuft (at). Note that the purple dots surrounding the apical tuft (at) are labeling artifacts. Position of the apical tuft (at) is indicated by sketch. Note that the 5-HT-LIR in the posterior larva is restricted to the midgut and the chaetal sacs. **d** At 7 dpf the apical circumesophageal connective (cc) shows distinct FMRFamide-LIR. Contour of the apical nerve ring (ar) is denoted (red dotted line), prominent perikarya are marked (red asterisk). **e** Lateral view of previous stage. Contour of the apical ring (ar) and the circumesophageal connective (cc) is denoted (dotted red line), prominent perikaryon is marked (red asterisk). **f** 5-HT-LIR at 7 dpf is present in the circumesophageal connective (cc), an esophageal nerve (en) with branching nerve fibers (eb) and the tissue underlying the esophagus (es). Note distinct 5-HT-LIR in at least 1 perikaryon (arrow). The connection of the latter perikarya with the esophageal nerve (en) is indicated by white dots. The inset shows the 5-HT-LIR (now grey) within the tissue underlying the esophagus in higher resolution. Notably, the signal underlying the esophageal region may also contain some unspecific staining due to the possible presence of gland cells in the esophagus. **g** At 14 dpf the 5-HT-LIR (and aTub-LIR) increases, but the pattern is comparable to the previous stage. No 5-HT-LIR is detectable apically (lack of signal indicated by red dotted circle). The inset shows a higher magnification of the tissue underlying the apical tuft. The purple dots surrounding the region of interest are labeling artifacts. Note that the overlap of the 5-HT-LIR and the aTub-LIR causes a white color in some parts of the shown image. The esophageal branching nerves are not shown in the image. **h** FMRFamide-LIR at 14 dpf is restricted to the apical ventral (vr) and dorsal root (dr) of the circumesophageal connective, the apical nerve ring and apical perikarya, and detectable in the tissue underlying the esophagus (white dotted circle). Inset shows volume rendering of FMRFamide-LIR. Position of the apical tuft (at) is indicated by sketch. Position of perikarya (red asterisks), apical nerve ring (ar, red dotted line) and roots of the circumesophageal connective (vr, dr, red dotted line) is indicated. Both roots of the ventral circumesophageal connective (vpvr, dpvr) are detectable by tubulin staining (dotted red line). Note that the overlap of the FMRFamide-LIR and the aTub-LIR causes a white color in some parts of the shown image. The inset shows the same volume rendered FMRFamide-LIR. The shape of neural structures is implemented by red dotted lines. ao, apical organ; ar, apical nerve ring; at, apical tuft; cb, ciliary band; cc, circumesophageal connective; ch, chaetae; cs, chaetal sac; dr, dorsal root of cc; dpvr, dorsal part of vr; eb, branches of the esophageal nerve; en, esophageal nerve; es, esophagus; gl, gland; mg, midgut; vr, ventral root of cc; vpvr, ventral part of vr. Scale bars = 30 μm (**a**), 100 μm (**b**), 15 μm (**d, e**), 50 μm (**f-h**). The scale bars only refer to the main images, not to the insets. **c** represents a more detailed picture of (**b**) and therefore has no scale bar

FMRFamide-like immunoreactivity (−LIR) occurs in 1 dpf specimens underneath the apical tuft (Figs. 3a, 5 and Additional file 1). A spherical region underlying the apical tuft is stained, but no distinct somata are yet detectable in this early stage. Although all investigated larvae show a comparable FMRF-amide-LIR underlying the apical tuft, unspecific staining cannot be excluded based on the data presented herein. In contrast 5-HT-like immunoreactivity (−LIR) is not detectable at this stage. That changes in the 48hpf (hours post fertilization) stage, where 5-HT-LIR clearly marks two prominent somata in the equatorial plane. From these somata circumesophageal nerves run apically, surround the esophagus and fuse underneath the apical tuft (Figs. 3b, c and 6). Additionally, 5-HT-LIR is detectable anterior to the latter connectives in the ventral esophageal tissue (Figs. 3c and 6). Appearance of immunoreactivity in the midgut lumen is likely a false positive signal caused by unspecific binding from the dense ciliation of the digestive tract. We regard expression of 5-HT within the gut lumen as unlikely. No FMRFamide-LIR apical signal could be detected at 48 hpf. But FMRFamide-positive apical signal is again present from 7 dpf onwards. Our experiments revealed distinct FMRFamide-LIR flask-shaped somata and a nerve ring underlying the apical tuft are present. Due to their position, these perikarya are regarded as part of a regular apical organ. In addition, the apical circumesophageal connective shows FMRFamide-LIR (Figs. 3d, e, 5 and Additional file 3). 5-HT-LIR is still solely exhibited in the circumesophageal connective, and the distinct somata at the equatorial plane and in the esophageal tissue (Figs. 3f, 6 and Additional file 2). Notably, within the esophagus the 5-HT-LIR is restricted to a prominent esophageal midline nerve running towards the anterior, in addition to distinct somata at the anterior end of the esophageal nerve and a dense meshwork of somata and nerves underlying the esophagus (Figs. 3f and 6). The latter structures also show prominent immunoreactivity against acetylated α-tubulin (aTub-LIR) (Figs. 5 and 6), whereas earlier stages only showed aTub-LIR in cilia. Such a developmental pattern with neuropeptide immunoreactivity arising early in the apical organ, the circumesophageal connectives and the anterior stomatogastric nervous system is well-known in other lophotrochozoans, such as the errant annelids *Platynereis dumerilli* [22] and *Phyllodoce groenlandica* [44], the sedentary annelids *Sabellaria alveolata* [20], *Capitella teleta* [9, 45] and *Scoloplos armiger* [25], sipunculids [21] and aplacophoran molluscs [46]. The absence of serotonergic somata in the apical organ of the early stages of *O. fusiformis* differs from the situation in most lophotrochozoans, such as Annelida, Mollusca, Entoprocta, and Platyhelminthes, where prominent apical flask-shaped neurons showing 5-HT-LIR (and FMRFamide-LIR) appear early in the development [20, 24, 46–49]. An apical absence of serotonergic somata has only been reported in Ectoprocta so far [50]. Moreover, the fact that a pair of 5-HT-LIR somata in the equatorial plane gives rise to the circumesophageal connectives is not described so far. These prominent cells could correspond to more apically situated 5-HT-LIR cells in other groups and that the spatial change towards posterior in *Owenia fusiformis* is owed by the peculiar morphology of the mitraria. As described for other annelids the circumesophageal connectives develop out of two prominent anterior perikarya by contralateral projection [26]. The two distinct somata in *O. fusiformis* might represent the same anterior perikarya after spatial shift. Whether this hypothesis turns out to be true needs further analysis, and additional investigations in other basally branching taxa are necessary to raise comparable data.

### Development of the late mitraria (14 dpf and older)

In later stages (14 dpf) the intensity of the aTub-LIR, 5-HT-LIR and FMRFamide-LIR increases along the circumesophageal connective, in the apical organ and within the esophageal tissue (Figs. 3g, h, 5, 6 and Additional file 5). Although additional FMRFamidergic somata are detectable in the apical organ, no distinct somata with 5-HT-LIR occur close to the apical tuft (Figs. 3g and 5). The circumesophageal connective splits into a dorsal and ventral root, both showing aTub-LIR. The ventral root furthermore provides a subdivision into two parts, a ventral and a dorsal one (Figs. 3h, 5 and 6). Unlike the dorsal root which exhibits both 5-HT-LIR and FMRFamide-LIR, the ventral root mainly shows aTub-LIR, and only the apical part before the subdivision in the ventral and dorsal part of the ventral root shows FMRFamide-LIR (Figs. 3g, h, 5 and 6). As shown for the previous stage, the stomatogastric nervous system at 14 dpf reveals clear 5-HT-LIR of the ventral most esophageal tissue, distinct anterior somata showing 5-HT-LIR and a prominent midline nerve with branching nerves running towards the ventral side (Figs. 3g and 6). A novel feature of this developmental stage is the appearance of FMRFamide-LIR in the ventral-most esophageal tissue (Figs. 3h and 5).

**Fig. 4:**
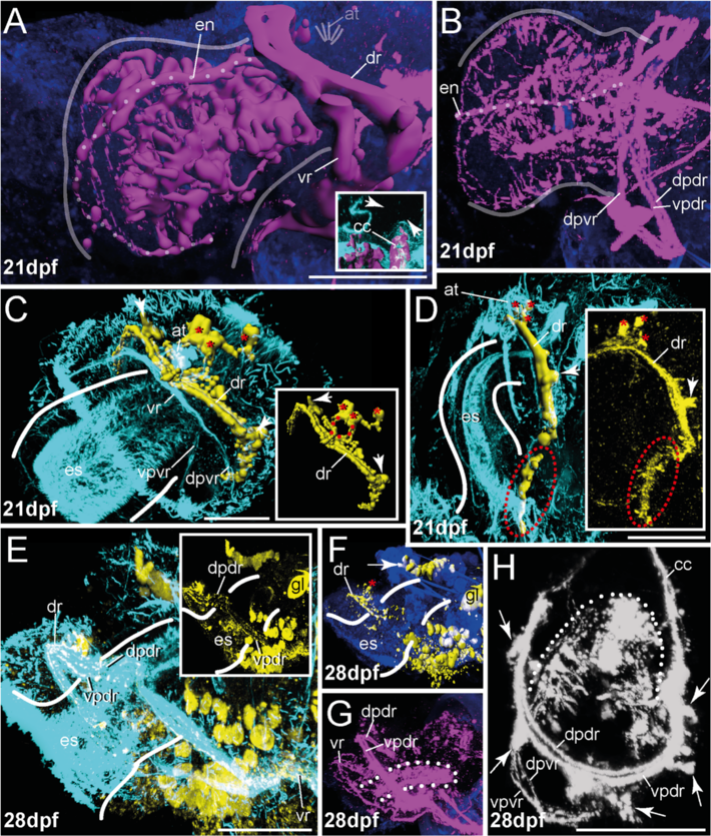
Innervation of apical organ and esophageal region in late developmental stages of *O. fusiformis* (21 dpf - 28 dpf). FMRFamide-LIR (*yellow*) and 5-HT-LIR (*purple*), aTub-LIR (*cyan*), DNA (*dark blue*)**.** Confocal z-projections and volume renderings of 5-HT-LIR (**a**; a, inset) (except of (**h**) where 5-HT-LIR is shown in *grey*)**,** FMRFamide-LIR (**c**, **d**, **f**) and aTub-LIR (**e**, inset). Ventral is left and apical is up in all images, except of (**b**) which shows a view from dorsal and (**h**) where the specimen is shown in antero-dorsal view with the anterior part down. Stages are given in days post fertilization (dpf). The shape of the esophagus is indicated by white lines. **a** At 21 dpf 5-HT-LIR is visible in the circumesophageal connective (cc) and its roots (vr, dr), within the esophageal nerve (en, dotted line) including all branching fibers and the tissue underlying the esophagus. Position of the apical tuft (at) is indicated by sketch. The inset shows absence of 5-HT-LIR in the apical region (indicated by white arrow heads). **b** The 5-HT-LIR frames the entire esophagus. Position of the esophageal nerve (en) is indicated by the dotted line. Note that -LIR is absent in the region of the apical organ. **c** Although both roots of the circumesophageal connective (cc) are detectable via anti-tubulin staining at 21 dpf, FMRFamide-LIR is restricted to the dorsal root (dr) including prominent lateral perikarya (arrow head), the apical nerve ring (red dotted line in inset), and the distinct apical perikarya (red asterisks). The inset shows the FMRFamide-LIR in the same view without background staining. The position of the apical ring is implicated by red dots. The distinct apical perikarya are marked (red asterisks). **d** A lateral view reveals prominent FMRFamide-LIR in the tissue underlying the esophagus (es) (red dotted circle). The inset shows the dorsal root of circumesophageal connective (dr) and the tissue underlying the esophagus (es) (red dotted circle) in confocal z-projection (with the same scale). Note the prominent lateral (arrow head) and apical perikarya (red asterisks). **e** At 28 dpf, approximately 1 week after metamorphosis, FMRFamide-LIR is can be detected within the dorsal root of the circumesophageal connective (dr), in the ventral nerve cord (vn) and numerous antero-ventral perikarya. Inset shows the FMRFamide-LIR of the prominent dorsal and ventral part of the dorsal root of the circumesophageal connective (vpdr, dpdr). Note that the overlap of the FMRFamide-LIR and the aTub-LIR causes a white color in some parts of the shown image. The inset shows only the FMRFamide-LIR. **f** FMRFamide-LIR reveals presence of the apical perikarya in juveniles (red asterisk) and shows a prominent dorsal assemblage of perikarya (arrow head). **g** 5-HT-LIR is exhibited within both roots (and the dorsal and ventral part of dr) of the circumesophageal connective (vpdr, dpdr, vr) and the tissue underlying the esophagus (dotted white line). Note that the pattern of -LIR in this tissue is invaginated compared to pre-metamorphic stages. **h** An antero-dorsal view reveals the presence of both parts of both circumesophageal roots (vpvr, dpvr, vpdr, dpdr) and perikarya (white asterisks) with 5-HT-LIR close to the roots. The tissue underlying the esophagus is detectable (dotted white line). Note that the pattern of -LIR in this tissue is invaginated compared to pre-metamorphic stages. at, apical tuft; cc, circumesophageal connective; dpdr, dorsal part of dr; vpdr, ventral part of dr; dpvr, dorsal part of vr; dr, dorsal root of cc; en, esophageal nerve; es, esophagus; gl, gland; vpvr, ventral part of vr; vr, ventral root of cc. Scale bars = 50 μm (**a–e**) and 30 μm (**h**). The scale bars only refer to the main images, not to the insets

**Fig. 5:**
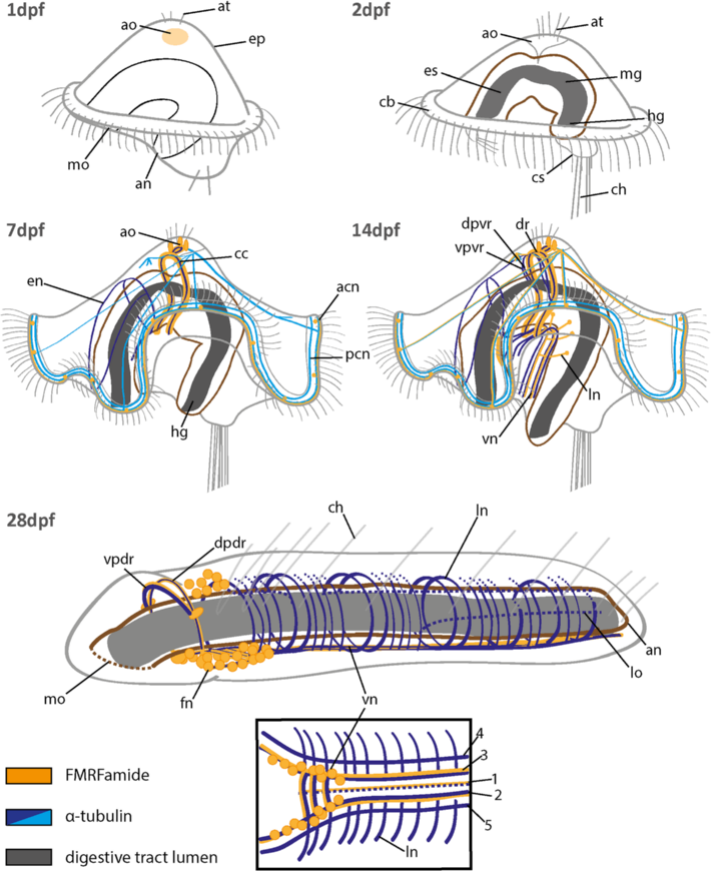
Schematic overview showing the pattern of FMRFamide-LIR and aTub-LIR during the development of *O. fusiformis.* Note that main neural patterns and the position of the digestive tract are color coded. Stages are given in days post fertilization (dpf). Ventral is left and apical is up in all images, except of the 28 dpf stage, where ventral is down. The inset shows a ventral view of the 28 dpf stage. The schemes presented here are simplified to support an understanding of the complex morphology. acn, apical ciliary ring nerve; an, anus; ao, apical organ; at, apical tuft; cb, ciliary band; cc, circumesophageal connective; ch, chaetae; cs, chaetal sac; dpdr, dorsal part of the dorsal root of cc; dpvr, dorsal part of the ventral root of cc; dr, dorsal root of cc; en, esophageal nerve; ep, episphere; es, esophagus; fn, FMRFamidergic neuron; hg, hindgut; ln, lateral nerve; lo, longitudinal nerve; mg, midgut; mo, mouth opening; pcn, posterior ciliary ring nerve; vn, ventral nerve cord; vpdr, ventral part of the dorsal root of cc; vpvr, ventral part of the ventral root of cc; vr, ventral root; 1–5, numbers referring to the specific nerves of vn

**Fig. 6:**
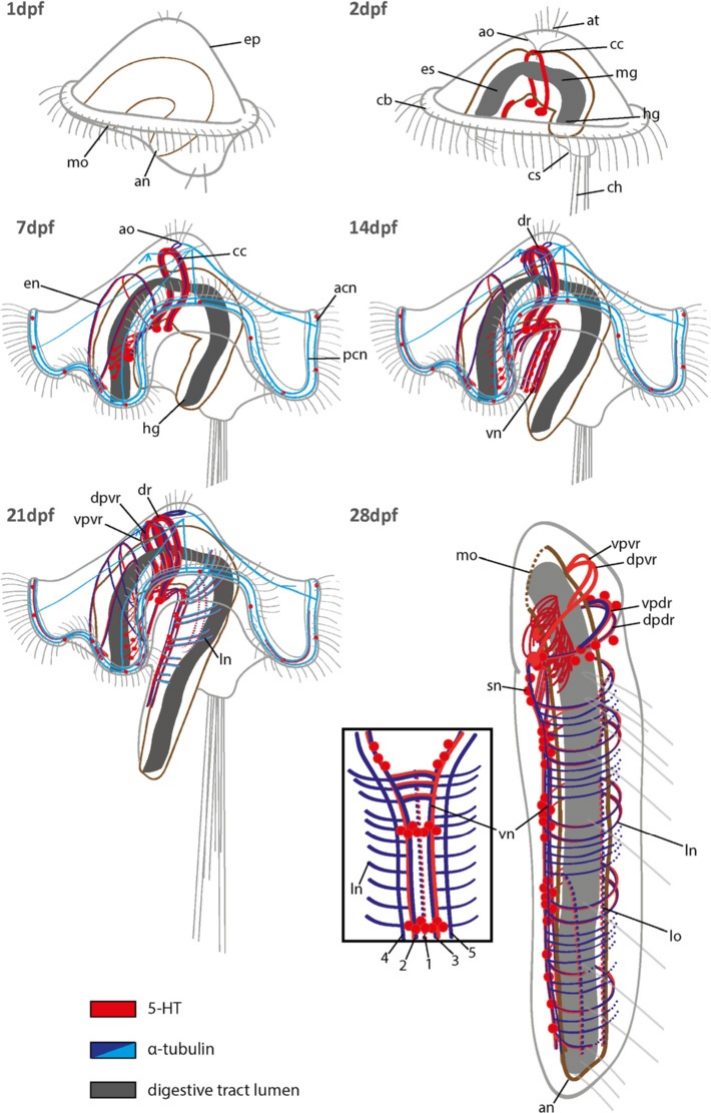
Schematic overview showing the pattern of 5-HT-LIR and aTub-LIR during the development of *O. fusiformis.* Note that main neural patterns and the position of the digestive tract are color coded. Stages are given in days post fertilization (dpf). Ventral is left and apical is up in all images, the inset shows a ventral view of the 28 dpf stage. The schemes presented here are simplified to support an understanding of the complex morphology. acn, apical ciliary ring nerve; an, anus; ao, apical organ; at, apical tuft; cb, ciliary band; cc, circumesophageal connective; ch, chaetae; cs, chaetal sac; dpdr, dorsal part of the dorsal root of cc; dpvr, dorsal part of the ventral root of cc; dr, dorsal root of cc; en, esophageal nerve; ep, episphere; es, esophagus; hg, hindgut; ln, lateral nerve; lo, longitudinal nerve; mg, midgut; mo, mouth opening; pcn, posterior ciliary ring nerve; sn, serotonergic neuron; vn, ventral nerve cord; vpdr, ventral part of the dorsal root of cc; vpvr, ventral part of the ventral root of cc; vr, ventral root; 1–5, numbers referring to the specific nerves of vn

At 21 dpf specimens feature all characteristics mentioned before, but all nervous system compartments become much more complex (Figs. 5 and 6). The stomatogastric nervous system increases in size with the esophagus, and in particular, the domains exhibiting 5-HT-LIR and aTub-LIR (Fig. 6) form a dense ventral meshwork of somata and interconnecting fibers (Fig. 4a and b) covering mainly the ventral part of the esophagus. The distinct esophageal midline nerve with branching nerves running towards the ventral shows 5-HT-LIR and aTub-LIR, with the anterior somata showing 5-HT-LIR and being present in this stage (Figs. 4a, b and 6). The FMRFamide-LIR at this region is comparable to younger stages, mentioned previously (Fig. 4d). The FMRFamide-LIR of the circumesophageal connective is still restricted to the dorsal root, but now two parts (ventral and dorsal) of the dorsal circumesophageal root are recognizable by staining against FMRFamide and α-tubulin (Figs. 4c and 5). The 5-HT-LIR occurs in both parts of the ventral and the dorsal esophageal root (Figs. 4a, b and 6). Given that the early developmental stages of other annelids also show a subdivision of both circumesophageal roots [23, 51], this trait, which clearly develops early during brain differentiation, is likely a plesiomorphic annelid feature [26]. Notably, the apical organ and the underlying nerve ring perpetuate in lacking any 5-HT-LIR signal, whereas FMRFamide-LIR is obvious in distinct somata and connecting fibers (Figs. 4c, d and 6). For comparison and a possible explanation of this unusual lack of 5-HT-LIR in the apical organ, please see section on development of the early mitraria (1 dpf-7 dpf).

### Post-metamorphic development

Shortly after metamorphosis (28 dpf) the dorsal root of the circumesophageal connective, consisting of a dorsal and a ventral part, still shows strong aTub-LIR and FMRFamide-LIR (Figs. 4e–g and 5). Furthermore, prominent somata appear, situated posterior to the mouth opening at the transition from the circumesophageal connective to the ventral nerve cord (Figs. 4e–g and 5). Forming a dense assemblage of perikarya, these FMRFamide-LIR somata frame the anterior end of the ventral nerve cord laterally (Figs. 4f and 5). Additionally, a distinct mass of FMRFamide-LIR somata is present dorsally, posterior to the circumesophageal connective (Figs. 4f and 5) and presumably belongs to the stomatogastric nervous system. 5-HT-LIR is present mainly in the dorsal and ventral root of the circumesophageal connective, although all subdivisions show some staining (Figs. 4h and 6). Notably, the ventral root consists of a dorsal and ventral part and frames the mouth opening which is only visible via anti-5-HT-staining. Furthermore several 5-HT-LIR somata are visible in close proximity to both major roots of the circumesophageal connective (Figs. 4h and 6).

The exhibited immunoreactivity of the esophageal nervous system is similar to the previous stage in regard to shape and composition (Figs. 4g, 5 and 6). But in contrast to earlier stages, the whole esophageal nervous system is inverted (together with the esophagus) and points inside the animal (Figs. 4g and 6). In earlier stages the immunoreactivity in the nerve fibers and perikarya underlies the esophagus and is detectable anterior to the circumesophageal connective which results in a distinct staining of the esophageal nerve with branching nerves and numerous antero-ventral oriented somata (see Fig. 4a and b). After metamorphosis the entire meshwork of fibers and somata that formerly underlied the larval esophagus anterior to the circumesophageal connective, is flapped backwards and can be detected posterior to the circumesophageal connective (Fig. 4g and h). This remarkable change in position and orientation of neural tissue is in line with the above described oral uptake of epidermal tissue during metamorphosis. We assume that this transformation goes along with a posteriorly directed folding of the esophageal nerve tissue. To which extent the uptake of epidermal tissue undergoes apoptosis, as assumed by Wilson [32], or whether it is reorganized into stomatogastric epidermis, needs further investigation.

At the former position of the larval apical organ, remnants of the FMRFamide-LIR nerve ring and FMRFamide-LIR somata are still present at the apical end of the dorsal circumesophageal root (Fig. 4f). Thus, an integration of apical organ cells into the adult brain can be verified. To what extent these cells become part of the adult nervous system cannot be determined by the present investigation. So far, investigations on different protostome lineages indicate a partial loss of the apical organ during or shortly after metamorphosis. Whether apical cells become part of the adult brain is still questionable [52–54]. Instead, investigations in *C. teleta* and *P. dumerilii* reveal retention of many larval neuronal structures in post-metamorphic stages, and therefore support the hypothesis of at least partial inclusion of apical larval somata into the adult nervous system [9, 22, 55]. Further detailed investigations on basally branching annelid taxa like *O. fusiformis*, including cell fate studies, would clarify this issue.

### Innervation of the ciliary band

**Fig. 7:**
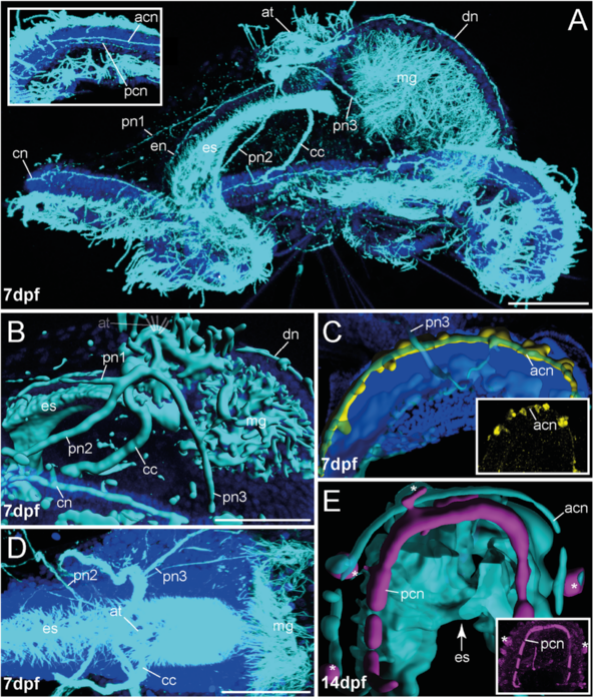
The innervation of the ciliary band in the mitraria of *O. fusiformis*. FMRFamide-LIR (*yellow*) and 5-HT-LIR (*purple*), aTub-LIR (*cyan*), DNA (*dark blue*). Confocal z-projections and volume renderings of aTub-LIR (**b**, **e**), FMRFamide-LIR (**c**) and 5-HT-LIR (**e**). Ventral is left and apical is up in all images, except (**c**) and (**e**) which show a view from ventral. Stages are given in days post fertilization (dpf). **a** The mitraria exhibits two distinct neurites forming the apical and posterior ciliary ring nerves (acn, pcn) (inset), and three pairs of peripheral nerves (pn1–3) running from apical towards ventral (pn1, pn2) and lateral (pn 3), and innervating the ciliary ring nerves (cn). Furthermore, a single prominent dorsal nerve (dn) runs from apical to the posterior ciliary ring nerve (pcn). The inset shows a higher magnification of the ciliary ring nerves. **b** The peripheral nerves (pn1–3) and the dorsal nerve (dn) interconnect the apical part of the developing embryo with the ciliary ring nerves (cn). **c** FMRFamide-LIR in the ciliary ring nerves is restricted to the apical ciliary ring nerve (acn), which also bears numerous FMRFamide-LIR perikarya. The inset shows a confocal z-projection of the same area in same orientation. Only the apical ciliary ring nerve (acn) is visible. This pattern is visible throughout the entire ring nerve also in stages 7 dpf - 21 pdf. **d** The peripheral nerves (pn1–3) and the dorsal nerve branch off from the circumesophageal connective (cc) at the apical pole of the larva. **e** 5-HT-LIR in the ring nerve is restricted to the posterior ciliary ring nerve (pcn) and mainly detected close to the esophagus (es). Note the perikarya showing 5-HT-LIR along the anterior ciliary ring nerve (*white asterisks*). The inset shows a confocal z-projection of the same area in same orientation. Only the posterior ciliary ring nerve (pcn) is visible. The staining shown here appeared to be fragmentary when looking at the entire ring nerve in stages 7 dpf - 21 pdf. acn, apical ciliary ring nerve; at, apical tuft; bn, bell nerve; cc, circumesophageal connective; cn, ciliary ring nerve; dn, dorsal nerve; en, esophageal nerve; es, esophagus; mg, midgut; pcn, posterior ciliary ring nerve; pn1–3, peripheral nerves 1–3. Scale bars = 50 μm. The scale bars only refer to the main images (**a, b, d**), not to the insets. Note that (**c**) and (**e**) are without scale

The innervation of the ciliary band in *Owenia fusiformis* is visible the earliest at 7d of development and is characterized by the presence of an apical and a posterior nerve underlying the prominent ciliary band (Figs. 5 and 6). These distinct nerves show aTub-LIR and 5-HT-LIR (in the posterior nerve) and FMRFamide-LIR (in the apical nerve) (Fig. 7a–e). Furthermore, both prominent nerves are interconnected to the remaining embryo by specific nerves running from the apical circumesophageal connective towards the ciliary band in a ventro-lateral direction or from the apical nerve ring in a dorsal direction (Fig. 7a, b, d; Additional file 4). An equatorial ciliary band with an underlying nerve possessing both 5-HT-LIR and FMRFamide-LIR and neurites interconnecting the latter structure with the apical nervous system, are described for various annelid larvae [9, 17–22, 24] and other trochophore larvae [46, 49, 56] and is named as prototroch. In these larvae the prototroch acts as an important effector organ that regulates larval swimming behaviour. Projections of apical primary sensory cells, as well as apical organ interneurons, travelling along the larval circumesophageal connectives to the ciliary ring nerve and to individual prototroch cells, have been shown to directly contribute to larval steering [12, 57]. In contrast to other annelids, the circumesophageal connectives of *O. fusiformis* do not pass close to the ciliary ring nerve, hence, in *Owenia* the side-branches of these connectives that go towards the ciliary ring are necessarily long neurites. This situation may be due to the above described lateral detachment of the epidermis from the central tissue complex. Furthermore, prominent ventral and dorsal nerves connecting an apical ring and the nerves underlying the ciliary ring also described for phyllodocid annelids [18, 24].

Thus, the presence and the characteristics of an underlying ciliary ring nerve and the comparable connection to the circumesophageal connectives indicate homology of the equatorial ciliary band of *O. fusiformis* with the prototroch of other annelids. Further comparative investigations like cell lineage studies on the origin of the ciliary band may be interesting in this context.

Beneath the prominent nerve fibers, the equatorial ciliary band possesses numerous unique somata with 5-HT-LIR and FMRFamide-LIR located in the entire ciliary band (Figs. 5, 7c and e). They may represent sensory organs, but detailed investigations are missing so far. After metamorphosis the ciliary band and the underlying nerve ring with the distinct somata disappear.

### Trunk nervous system

#### Development of the late mitraria (14 dpf and older)

**Fig. 8:**
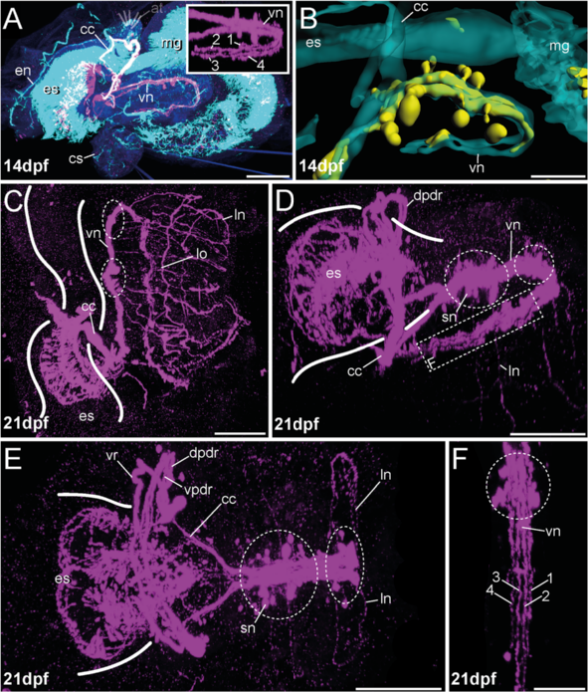
Development of the trunk nervous system in different pre-metamorphic developmental stages of *O. fusiformis* (14 dpf–21 dpf). FMRFamide-LIR (*yellow*) and 5-HT-LIR (*purple*), aTub-LIR (*cyan*), DNA (*dark blue*)**.** Confocal z-projections and volume renderings of FMRFamide-LIR (**b**) and aTub-LIR (**b**). Ventral is left and apical is up in (**a**) and (**b**), in (**c**) ventral is down and apical is left, (**e**) and (**f**) which show a view from apical with the ventral side left (**e**) and down (**f**). **d** shows a view from latero-apical with the ventral side left. Stages are given in days post fertilization (dpf). The shape of the esophagus (and intestine) is indicated by white lines. The position of somata clusters showing 5-HT-LIR is indicated with white dotted circles. **a** 5-HT-LIR of the trunk nervous system is detectable earliest at 14 dpf. The ventral nerve cord (vn) is represented by four neurites including numerous serial perikarya (inset). Note that the overlap of the 5-HT-LIR and the aTub-LIR causes a white color of the circumesophageal connective (cc). The inset shows an apical view of the ventral nerve cord (vn). **b** FMRFamide-LIR at 14 dpf is restricted to the two inner parallel neurites and four pairs of perikarya. **c** At 21 dpf, shortly prior to metamorphosis, the 5-HT-LIR of the ventral nerve cord (vn) is detectable within distinct neurites, serially arranged clusters of somata (white dotted circle) and serial lateral nerves (ln) showing 5-HT-LIR and branching off from the serotonergic cell clusters. Notably, numerous longitudinal nerves (lo) are visible in pre-metamorphic stages. The specimens represent conditions later on observable in juveniles as well. aTub-LIR was hardly detectable due to a dense ciliation of the entire specimen. **d** A lateral view at 21 dpf reveals several clusters of somata exhibiting 5-HT-LIR. Several lateral nerves (ln) branch off from the ventral nerve cord (vn) at the position of the cell clusters. Note that the posterior part of the ventral cord (vn) is bent under the anterior part and the esophageal region (es) due to the shape of the mitraria. The dotted square indicates the region that is shown in (**f**) with higher magnification. **e** An apical view at 21 dpf reveals the presence the dorsal and the ventral parts of the dorsal root of the connective (vpdr, dpdr) and the ventral root of the connective (vr). Furthermore, distinct clusters of serotonergic neurons (sn) along the ventral nerve cord (vn) are detectable. Prominent lateral nerves (ln) branch off from these clusters. The posterior part of the ventral cord (vn) is bent under the anterior one and therefore not visible. **f** A higher magnification of the posterior part of the ventral nerve cord (vn) at 21 dpf reveals the presence of 4 distinct nerves showing 5-HT-LIR (numbered 1–4). The location of the posterior ventral nerve cord (vn) in the entire animal is shown in (**d**) and marked with the white dotted square. at, apical tuft; cc, circumesophageal connective; cs, chaetal sac; dpdr, dorsal part of the dorsal root of cc; en, esophageal nerve; es, esophagus; gl, gland; hg, hindgut; ln, lateral nerve; lo, longitudinal nerve; mg, midgut; vn, ventral nerve cord; vpdr, ventral part of the dorsal root of cc;vr, ventral root of the cc. Sale bars = 100 μm (**a**), 30 μm (**b**), 50 μm (**c–e**) and 20 μm (**f**). The scale bars only refer to the main images, not to the insets

The ventral nervous system of the trunk is detectable via antibody staining not earlier than 14 dpf (Figs. 5, 6, 8a, b; Additional file 6 and Additional file 7). Approaches to stain the trunk nervous system in earlier stages remained without result. Originating at the base of the circumesophageal connective, two parallel bundles of two nerves run individually towards the pygidium, showing -LIR for FMRFamide, 5-HT and aTub-LIR (Figs. 5, 6 and 8a, b). FMRFamide-LIR is present just in the ventral cord and in four pairs of lateral somata interconnected with the cord (Figs. 5 and 8b), whereas 5-HT-LIR is obvious in the entire ventral cord including numerous serially arranged somata covering the entire ventral cord (Figs. 6 and 8a). Thus, the ventral cord contains two pairs of nerves possessing 5-HT-LIR, whereas only one pair can be counterstained against FMRFamide (Figs. 5, 6 and 8a, inset; b). Notably, both the FMRFamide-LIR and 5-HT-LIR is extending in an antero-posterior direction with increasing age of the developmental stages. None of the -LIR was firstly detected posteriorly. These finding are in accordance with findings from several errant and sedentary annelids [8, 17, 20, 23, 58, 59], as well as from sipunculids [21] and other lophotrochozoan taxa, e.g., the putative closely related Solenogastres (Mollusca) [46] and Polyplacophora (Mollusca) [60, 61]. In contrast to other investigated annelids, such as some serpulids and phyllodocids, 5-HT-LIR (but not FMRFamide-LIR) shows up first at the posterior end of the larva and extends anteriorly [19, 24, 26]. To what extent the described differences correlate with the absence or presence of specific neurons and other larval characteristics like yolk contents, and the significance of these differences for axonal pathfinding and nerve cord differentiation, is a matter of debate and probably needs further investigation. The presence of posterior 5-HT-LIR in the investigated mollusc representatives and the absence of posterior 5-HT-LIR in early stages of *O. fusiformis,* indicate that the latter pattern evolved within annelids. At 21 dpf, shortly before metamorphosis, the ventral nervous system in *O. fusiformis* increases in length due to larval growth but the pattern described for the previous stage remains comparable (Figs. 5, 6 and 8c–e). Notably, the ventral cord now consists of several (at least four) longitudinal nerves (Fig. 8f) and gives rise to numerous serial lateral nerves exhibiting 5-HT-LIR and aTub-LIR (Figs. 6 and 8c–f). The FMRFamide-LIR is comparable to that investigated in the 14 dpf-stage (not shown).

### Post-metamorphic development

**Fig. 9:**
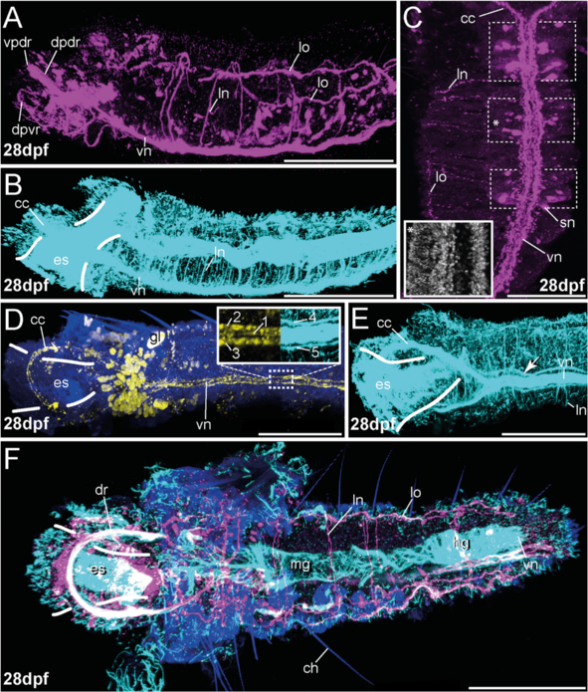
Development of the trunk nervous system in post-metamorphic juveniles of *O. fusiformis* (~28 dpf). FMRFamide-LIR (*yellow*) and 5-HT-LIR (*purple*), aTub-LIR (*cyan*), DNA (*dark blue*) (except for inset (C) where DNA staining is shown in grey). Confocal z-projections. Anterior is left in all images, except of (**c**) where anterior is up. **a** and **b** are lateral views, (**c**–**e**) are views from ventral, (**f**) is a view from dorsal. Stages are given in days post fertilization (dpf). The shape of the esophagus (and intestine) is indicated by white lines. The position of somata clusters showing 5-HT-LIR is indicated with white dotted rectangles. **a** Shortly after metamorphosis, at 28 dpf, 5-HT-LIR is present in the ventral nerve cord (vn), in numerous lateral nerves (ln) and prominent longitudinal nerves (lo). **b** Using the same specimen and orientation as in (A), the aTub-LIR reveals numerous lateral nerves (ln). **c** A ventral view at 28 dpf reveals serial clusters of somata (with 5-HT-LIR) and lateral nerves branching off from the ventral cord (vn) at the position of the cell clusters. The inset shows the region of the 2nd cluster of somata possessing 5-HT-LIR (marked with white asterisk in (C)) stained with DAPI. Notably, DNA staining does not show any cluster. **d** The ventral nerve cord (vn) is represented by two parallel neurites and a median nerve with FMRFamide-LIR (see left inset, nerves are labeled 1–3) and two outer parallel nerves only showing aTub-LIR (see right inset, nerves are labeled 4 and 5). At least nerves 2 and 3 arise in 5-HT staining as well (not shown). Nerve 1 is visible in some 5-HT stainings only. Notably, no serial clusters of somata showing FMRFamide-LIR are detectable along the ventral cord (vn). **e** aTub-LIR at 28 dpf reveals a pair of lateral longitudinal nerves (arrow head) running besides the main cord, and numerous lateral nerves (ln) leading towards the periphery. **f** 5-HT staining at 28 dpf shows serially arranged lateral nerves (ln) and numerous lateral perikarya. Furthermore, longitudinal nerves (lo) are running in antero-posterior direction. Note that the overlap of the 5-HT-LIR and the aTub-LIR causes a white color of the dorsal root of the circumesophaegal connective (dr). at, apical tuft; cc, circumesophageal connective; ch, chaetae; dr, dorsal root of cc; dpdr, dorsal part of the dorsal root of cc; dpvr, dorsal part of the ventral root of cc; en, esophageal nerve; es, esophagus; gl, gland; hg, hindgut; ln, lateral nerve; lo, longitudinal nerve; mg, midgut; sn, serotonergic neuron; vn, ventral nerve cord; vpdr, ventral part of the dorsal root of cc. Sale bars = 100 μm (A, B, D, E, F) and 50 μm (C). The scale bars only refer to the main images, not to the insets

In contrast to adults, where two parallel ventral cords fuse within the first segment and form a single ventral longitudinal nerve cord in *O. fusiformis* [27], the post-metamorphic trunk nervous system is first characterized by the presence of various distinguishable longitudinal nerves (Figs. 5, 6 and 8d–f). The ventral nerve cord is composed of two inner neurites exhibiting FMRFamide-LIR, 5-HT-LIR and aTub-LIR (Fig. 9d, inset; 5; 6), and two strands possessing only aTub-LIR laying lateral to the main cord –LIR (Fig. 9d, inset; 5; 6). Furthermore, a median nerve is visible showing FMRFamide-LIR (Fig. 9d, inset; 5). 5-HT-LIR/aTub-LIR was hardly detectable for this nerve in our stainings, but its presence cannot be excluded (Figs. 5 and 6a). A ventral nerve cord transiently comprising five longitudinal nerves including a median nerve during development is thought to represent an ancestral feature of annelids [26, 27] and can be observed in *Capitella teleta*, as well [9]. The presence of this pattern in post-metamorphic stages of a basally branching annelid species clearly supports this hypothesis.

Another remarkable feature of the investigated post-metamorphic animals is the presence of serially arranged clusters of somata that show 5-HT-LIR and which are suggestive of ganglion-like assemblages, and numerous branching lateral nerves with 5-HT-LIR/ aTub-LIR arranged in a similar serial pattern (Figs. 9a–c, f and 6). Notably, additional lateral nerves can be observed, which show only aTub-LIR and do not originate from the somata clusters showing 5-HT-LIR (Fig. 9b and e). The FMRFamide-LIR somata are limited to the anterior end of the ventral cord and do not exhibit seriality (Fig. 9d; 5). The observed serial patterns of neural structures reflect the external body segmentation of the juvenile worms and resemble the conditions known for most other annelids including *C. teleta* and *P. dumerilii* [9, 22, 26, 27]. Whereas no such serial clusters of somata are described for adult *Owenia fusiformis* [34, 37], immunohistochemical investigations in *Galathowenia oculata* revealed the presence of comparable structures only in few posterior-most segments of adult worms [38]. The early serial patterns of neural structures within the entire ventral nerve cord as described for *Owenia fusiformis* are not detectable in later ontogenetic stages, a situation that is well described for annelid taxa without adult external body segmentation, such as sipunculans, echiurans or myzostomids [17, 21, 62, 63]. Thus, the lack of ventral nerve cord ganglia in adult *Owenia fusiformis* differs from the situation in other annelids, but the serial arrangement of at least certain neural structures in juvenile stages as described above might be well comparable with the nervous system architecture known from other taxa.

## Conclusions

Our analyses of neurogenesis in different developmental stages of *Owenia fusiformis* obviously show that the metamorphosis of the enigmatic mitraria larva to the juvenile is not that catastrophic as previously thought. Instead, an antero-posterior development of neural structures showing prominent 5-HT-LIR, FMRFamide-LIR and aTub-LIR is detectable, and adult nervous system precursors are present in early stages of development. The drastic changes in body shape during metamorphosis occur mainly by diminution of the larval blastocoel and rearrangement of the detached epidermis of the bell-shaped swimming larva. Our investigations on the development of the nervous system reveal also many similarities to other annelids throughout all developmental stages. Thus, adult precursors are present in early stages and juveniles. The same developmental stages exhibit a prominent apical organ formed by flask-shaped perikarya, early development of circumesophageal connectives interconnecting apical and trunk nervous system, and the serially arranged clusters of somata displaying 5-HT-LIR in the ventral nerve cord in addition to the serial lateral nerves (at least in early juveniles). These highly comparable features are also known among basally branching and deeply nested annelid groups and are described for other Lophotrochozoa such as mollusks. It will be an interesting to study, whether all neuronal somata are clustered and whether they form ganglion-like structures during early development in basally branching annelids as in *Owenia fusiformis*. However, we are not in position to infer this from our current data. We observed a high number of lateral nerves showing only aTub-LIR not following a serial pattern, but the position of the respective somata remains unclear. Furthermore, the presence of an equatorial ciliary band including a distinct underlying nerve ring possessing 5-HT-LIR and FMRFamide-LIR and its connection to the circumesophageal connectives are features well known for annelid larvae. Notable differences include the absence of 5-HT-LIR in early larvae, the lack of a first prominent 5-HT-LIR at the posterior pole, an equatorial ciliary band that bears numerous distinct putative sensory cell clusters and the serial clusters of immunoreactive somata of the juvenile nervous system that disappear in older stages. Such annelid features are only known for *Owenia fusiformis* so far, but should be examined in other basally branching annelid groups, such as Magelonidae. Such comparative investigations are necessary for revealing the polarity of annelid trait evolution. With its comparatively simple brain consisting of relatively few somata (we here refer to the roots of the circumesophageal connectives as ‘brain’, because no other distinct brain elements were detected so far) and intraepidermal trunk nervous system, which bears distinct differences between juveniles and adults in terms of centralization and somata organization. Therefore, *O. fusiformis* offers great potential as a study object for unraveling the architecture and neural patterning mechanisms of the ancestral annelid nervous system. A deeper knowledge of these issues could yield further important insights into bilaterian developmental topics.

## Methods

### Animal culture and fixation

Adult specimens of *Owenia fusiformis* were collected in Saint-Efflam during summer 2013–15 (Brittany/ France), transferred to Bergen (Norway) and reared in a tempered sea-water cycle. After artificial fertilization in filtered sea water (FSW) the developmental stages were reared at 18 °C in glass flasks containing 1 l FSW. The culture was aerated, set under strict diurnal rhythm (14:10 – light: dark) and fed with a mix of unicellular algae (*Isochrysis*). Water was changed regularly.

Prior to fixation different larval stages were anaesthetized using 7 % MgCl_2_ in FSW. The larvae were then fixed in 4 % paraformaldehyde (PFA) in 1x phosphate buffered saline with Tween (1x PBS: 0.05 M PB / 0.3 M NaCl / 0.1 % Tween20) for 2 h at 4 °C. After fixing, the animals were rinsed in 1x PBS several times and stored in 1x PBS containing 0.05 % NaN_3_ at 4 °C until usage.

For semi-thin sections the specimens were fixed in 2.5 % glutaraldehyde in 1x PBS (0.05 M PB, 0,3 M NaCl) for 1 h at 4 °C and subsequently washed several times with PBS buffer over the next 24 h on 4 °C before stored in 1x PBS containing 0.05 % NaN_3_ at 4 °C until usage. For postfixation specimens were treated with 2 % Osmium tetroxide in 1x PBS for 20–40 min at 4 °C and subsequently dehydrated in a graded series of acetone/PBS at room temperature, transferred to propylenoxide and finally embedded using TAAB Araldite 502/812 Kit according to manufacturer’s recommendations.

Semi-thin sections were made from specimens at 7 dpf with a Leica EM UC7 ultramicrotome using a Diatome ultra jumbo diamond knife. Sections were transferred to glass slides, stained with toluidine blue (1 % toluidine blue, 1 % sodium tetraborate and 20 % sucrose) and mounted with Depex. Light microscopic images were taken with a Zeiss Imager Z2 microscope and a Zeiss Axiocam 506 color camera. Images were processed and image stacks were registered with Fiji. The final panels were designed using Adobe (San Jose, CA, USA) Photoshop CC and Illustrator CC.

For electron microscopy animals were treated as described in [64]. Accordingly, they were fixed in 2.5 % glutaraldehyde/ 0.1 M sodium cacodylate/ 0.24 M NaCl and subsequently post-fixed in 1 % OsO4/ 0.1 M sodium cacodylate/ 0.24 M NaCl. Specimens were then en bloc stained for 30 min in 2 % OsO4/ 1.5 % potassium ferricyanide/ 0.1 M sodium cacodylate followed by incubation in 2 % aqueous uranyl acetate for 30 min. Dehydration of the samples was performed gradually in ethanol series and then with propylene oxide. All steps were done at room temperature. Following embedding (using the TAAB Araldite 502/812 kit), ultrathin sections (70 nm) were cut with a Leica EM UC7 and counterstained with 2 % uranyl acetate and lead citrate. Images were acquired on a JEOL 1011 transmission electron microscope equipped with an Olympus MORADA camera. The final panels were prepared using Adobe (San Jose, CA, USA) Photoshop CC and Illustrator CC.

### Immunohistochemistry and confocal laser scanning microscopy

Anatomical details of developmental stages of *Owenia fusiformis* Delle Chiaje, 1844, were revealed in whole animal preparations using standard immunohistochemical staining protocols and a range of well-established antisera as neural markers. Every staining was carried out using at least 25–30 specimens of each stage. Although the specificities of the employed antibodies have all been established in numerous invertebrates (details below), we cannot fully exclude that a given antiserum may bind to a related antigen in the investigated specimens. We hence refer to observed labelled profiles as exhibiting (antigen-) like immunoreactivity (−LIR). Negative controls were obtained by omitting the primary antibody in order to check for antibody specificity and yielded no fluorescence signal. For immunohistochemistry specimens were rinsed 2 x 5 min in PTW (PBS with 0.1 % Tween 20) at RT (room temperature) and transferred into 10 μg proteinase K/ml PTW for 2–3.5 min depending on the developmental stage (24hpf-3dpf = 90s; 7 dpf = 2 min; 14–21 dpf = 2,5 min; after metamorphosis = 3,5 min). After 2 short rinses in glycine (2 mg glycine/ml PTW), and 3x 5 min washes in PTW, the specimens were re-fixed using 4 % PFA in PBS containing 0.1 % Tween for 20 min at RT. Subsequently the developmental stages were rinsed 2x5 min in PTW, 2x5 min in THT (0,1 M TrisCl, 0,1 % Tween) and blocked for 1-2 h in 5 % sheep serum in THT according to the protocol of Conzelmann and Jekely (2012) [65]. The primary antibodies, polyclonal rabbit anti-serotonin (INCSTAR, Stillwater, USA, dilution 1:500), monoclonal mouse anti-acetylated α-tubulin (clone 6-11B-1, Sigma-Aldrich, St. Louis, USA, dilution 1:500), and polyclonal rabbit anti-FMRFamide (ImmunoStar Inc., Hudson, USA, dilution 1:1000) were applied for 24–72 h in THT containing 5 % sheep serum at 4 °C. Specimens were then rinsed 2x 10 min in 1 M NaCl in THT, 5x 30 min in THT and incubated subsequently with secondary fluorochrome conjugated antibodies (goat anti-rabbit Alexa Fluor 488, Invitrogen, USA, dilution 1:500; goat anti-mouse Alexa Fluor 633, ANASPEC, Fremont, USA, dilution 1:500) in THT containing 5 % sheep serum for 24 h at 4 °C. Subsequently, the samples were washed 6x 30 min in THT, stained with DAPI for 10–15 min (5 mg/ml stock solution, working solution: 2 μl in 1 ml THT – final concentration 10 μg/ml) and washed 2x 5 min in THT. For clsm-analyses samples were mounted on glass slides using 90 % glycerol/10 % 10x PBS containing DABCO. Specimens were analyzed with the confocal laser-scanning microscope Leica TCS STED (Leica Microsystems, Wetzlar, Germany). Confocal image stacks were processed with Leica AS AF v2.3.5 (Leica Microsystems), ImageJ and Imaris 6.3.1 (Bitplane AG, Zurich, Switzerland). The final panels were designed using Adobe (San Jose, CA, USA) Photoshop CC and Illustrator CC.

## Abbreviations

5-HT, 5-Hydroxytryptamin (Serotonin); aTub, acetylated-α-tubulin; dpf, days post fertilization; FMRFamide, the neuropeptide Phe-Met-Arg-Phe; −LIR, −like immunoreactivity

## Additional files

Additional file 1: Confocal z-stack showing FMRFamide-LIR (purple), aTub-LIR (cyan) and DAPI staining (dark blue) of the 1 dpf stage. Lateral view. Note that the staining of peripheral nerves is scarce. Available at: http://dx.doi.org/10.5061/dryad.m8k24/1. Additional file 2: Confocal z-stack showing 5-HT-LIR (purple), aTub-LIR (cyan) and DAPI staining (dark blue) of the 7 dpf stage. Apical view. Note that the staining of peripheral nerves is scarce. Available at: http://dx.doi.org/10.5061/dryad.m8k24/2. Additional file 3: Confocal z-stack showing FMRFamide-LIR (purple), aTub-LIR (cyan) and DAPI staining (dark blue) of the 7 dpf stage. Apical view of the apical organ. Available at: http://dx.doi.org/10.5061/dryad.m8k24/3. Additional file 4: Confocal z-stack showing aTub-LIR (cyan) and DAPI staining (dark blue) of the 14 dpf stage. Lateral view. Available at: http://dx.doi.org/10.5061/dryad.m8k24/4. Additional file 5: Confocal z-stack showing FMRFamide-LIR (purple), aTub-LIR (cyan) and DAPI staining (dark blue) of the 14 dpf stage. Apical view. Note that the staining of peripheral nerves is scarce. Available at: http://dx.doi.org/10.5061/dryad.m8k24/5. Additional file 6: Confocal z-stack showing FMRFamide-LIR (purple), aTub-LIR (cyan) and DAPI staining (dark blue) of the 14 dpf stage. Lateral view. Note that the staining of peripheral nerves is scarce. Available at: http://dx.doi.org/10.5061/dryad.m8k24/6. Additional file 7: Confocal z-stack showing 5-HT-LIR (purple), aTub-LIR (cyan) and DAPI staining (dark blue) of the 14 dpf stage. Lateral view. Note that the staining of peripheral nerves is scarce. Available at: http://dx.doi.org/10.5061/dryad.m8k24/7.
